# Machine learning driven methodology for enhanced nylon microplastic detection and characterization

**DOI:** 10.1038/s41598-024-54003-1

**Published:** 2024-02-12

**Authors:** Cihang Yang, Junhao Xie, Aoife Gowen, Jun-Li Xu

**Affiliations:** School of Biosystems and Food Engineering, University College Dublin, Belfield, Dublin 4, Ireland

**Keywords:** Environmental sciences, Optics and photonics, Natural hazards

## Abstract

In recent years, the field of microplastic (MP) research has evolved significantly; however, the lack of a standardized detection methodology has led to incomparability across studies. Addressing this gap, our current study innovates a reliable MP detection system that synergizes sample processing, machine learning, and optical photothermal infrared (O-PTIR) spectroscopy. This approach includes examining high-temperature filtration and alcohol treatment for reducing non-MP particles and utilizing a support vector machine (SVM) classifier focused on key wavenumbers that could discriminate between nylon MPs and non-nylon MPs (1077, 1541, 1635, 1711 cm^−1^ were selected based on the feature importance of SVM-Full wavenumber model) for enhanced MP identification. The SVM model built from key wavenumbers demonstrates a high accuracy rate of 91.33%. Results show that alcohol treatment is effective in minimizing non-MP particles, while filtration at 70 °C has limited impact. Additionally, this method was applied to assess MPs released from commercial nylon teabags, revealing an average release of 106 particles per teabag. This research integrates machine learning with O-PTIR spectroscopy, paving the way for potential standardization in MP detection methodologies and providing vital insights into their environmental and health implications.

## Introduction

Since the 1950s, the mass production of plastics has been integral to industries such as medicine, construction, and clothing, leading to increased plastic waste and the generation of microplastics (MPs), defined as particles less than 5 mm in size^1,2^. MPs have been widely detected in our environment, food, and even in some important organs and tissues of the human body^3–5^. However, our understanding of the potential health risks associated with MPs is still limited. The toxicity of MPs is closely related to factors such as their abundance, size, and shape^6^; hence, a comprehensive understanding of these factors is required to better assess and the current lack of a standardized methodology in MP detection, inconsistencies exist in the reported number of MPs released across different laboratories using the same (or similar) plastic product as experimental material, which results in inaccurate assessment of MP toxicity. Research on MPs released from teabags is a compelling example to illustrate this. Teabags are considered a confirmed pathway of human exposure to MPs. However, the existing literature on the quantification of MPs released from teabags exhibits considerable variability and inconsistency (see Table 1). These discrepancies in the reported counts/quantities pose a significant barrier in assessing the potential hazards of MPs released from teabags.

**Table 1:** Summary of reported counts/quantities of MPs released from teabags.

Authors	Teabag material	Steeping condition	Quantification method	Results
Hernandez et al.^7^	Nylon teabag, PET teabag	5 min at 95 °C	Counting based on scanning electron microscopy images	11.6 billion MPs and 3.1 billion nano plastics/teabag
Busse et al.^8^	Nylon teabag	5 min at 95 °C	Particle-based analysis using Raman spectroscopy	5800–20,400 MPs/teabag
Ouyang et al.^9^	Nylon teabag, PET teabag	1 h at 100 °C	Particle-based analysis using FTIR spectroscopy	393 MPs/teabag (based on extrapolation of data reported by the authors)
Afrin et al.^10^	–	5 min at 95 °C	Particle-based analysis using FTIR spectroscopy	60 MPs/kg of teabags
Cella et al.^11^	Nylon teabag	10 min at 95 °C	Mass estimation based on Beer's law and FTIR spectral analysis	1.13 ± 0.07 mg of nano plastics/teabag
Wang et al.^12^	PLA teabag	30 min at 95 °C	Depolymerization followed by Liquid Chromatography–Tandem Mass Spectrometry analysis	12 μg of MPs/teabag

*PET* polyethylene terephthalate, *PLA* polylactic acid, *FTIR* Fourier-transform infrared spectroscopy.

A n-dash indicates the relevant information is unavailable.

Further investigation of these studies revealed several key factors that might have contributed to the observed inconsistencies. (1) Differences in brewing time and temperature. (2) The implementation or omission of particle-based analysis. Particle-based analysis entails qualitatively assessing each observed particle before quantification, which is essential for obtaining accurate measurements of MP quantities. Without this analysis, there is a risk of false positives (i.e., mistaking non-MP particles for MP particles). In the study by Hernandez et al.^7^, particle-based analysis was not conducted, therefore, Busse et al.^8^ critically commented that a significant portion of non-MP substances was erroneously identified as MP particles, contributing to the astonishing reported numbers. (3) Differences in instrument detection limits. Micro-Fourier transform infrared (micro-FTIR) and micro-Raman techniques can provide accurate chemical information about particles, enabling reliable particle-based analysis. However, due to variations in the detection limits of these two techniques, with micro-FTIR typically being > 10 μm and micro-Raman being > 1 μm, the particle counts obtained from these two techniques differ^8,9^. (4) Variation in teabags. Teabags could vary in material, size, weight, polymerization degree, and overall quality, which might have resulted in varying amounts of MPs released under similar conditions^13^. Future research associated with MPs released from teabags should consider the aforementioned factors to enhance the comparability of results in this theme. It is understood that factors related to detection limits and brewing time/temperature are largely dependent on the instrumentation available to the researcher and the research questions of interest. However, at the very least, particle-based analysis should be performed for accurate quantifications.

Improving the efficiency of MP analysis is also a critical step in accurately assessing and describing MP hazards^14^. Currently, a multitude of advanced and highly efficient techniques have emerged and been applied in MP research. Some notable instances are rooted in the application of quantum cascade lasers (QCLs). Due to the high intensity and high brilliance of QCLs, instruments using QCLs as IR radiation sources can rapidly acquire spectral data with high signal-to-noise ratios (SNR)^15^, thereby rendering swift and reliable MP analysis feasible. Two QCL-based approaches have been reported to be highly efficient in MP research. One couples mid-IR QCLs with a large focal plane array (FPA) detector, enabling the hyperspectral imaging (HSI) of a 144 mm^2^ area within 36 min^16^. Another employs mid-IR QCLs in tandem with a single-element detector and an intelligent automated MP analysis algorithm (software), thereby accomplishing the characterization and identification of a single particle within a 6–9 s timeframe, automatically^14^. In contrast, despite employing mid-IR QCLs as an IR source, the optical photothermal infrared (O-PTIR) technique has not demonstrated comparable efficiency to the aforementioned approaches. The O-PTIR technique offers submicron-level imaging resolution, artifact-free spectra, and minimizes the need for extensive sample preparation^17^, establishing it as a reliable and convenient tool in MP analysis. However, to advance the practical application of the O-PTIR technique, enhancing its efficiency becomes imperative. Strategies to achieve this aim encompass the development of a (large) FPA detector tailored for O-PTIR microscopes (presently employing only a single-element detector) and the formulation of automated MP analysis software^14^. Whereas the preceding two methods require prolonged research, harnessing the tunability of QCLs offers a straightforward and expedient route. In essence, owing to the use of QCLs, the O-PTIR technique enables the locking of IR radiation at a predetermined wavenumber (frequency), allowing for the selective collection of data solely from informative wavenumbers (e.g., discrete frequency infrared imaging, DFIR). This strategic approach can effectively truncate data collection duration (potentially from weeks down to hours) and hence significantly heightens efficiency.

The primary aim of this work is to propose an enhanced method for MP analysis integrating O-PTIR spectroscopy with machine learning algorithms, aiming to achieve reliable results and address inconsistencies in the existing literature. To achieve this, pre-treatment strategies including high-temperature filtration and alcohol treatment were employed to reduce the interference from non-MP particles. A machine learning model utilizing key wavenumbers was developed to efficiently predict each particle as MP or non-MP, resulting in faster and more accurate particle identification. This approach significantly reduces analysis time compared to correlation-based methods, making it highly applicable for handling large datasets in particle-based analysis.

## Results & discussion

### Contamination level and representativeness of subsampled areas

Analysis of the procedural blank sample indicated that contamination from the experiment environment was low. The detailed results are summarized in Table S1.

The positive control sample was used to examine the representativeness of the nine subsampled regions, determined by the ratio of the estimated mass of particles on the filter to the initial 0.05 mg of nylon microspheres. It was found that the method slightly overestimated the mass of nylon microspheres, with an obtained ratio of 1.27 ± 0.06. (Detailed information is displayed in Fig. S1). In an endeavour to optimize the number of regions for our spectral imaging model, we systematically analyzed the ratio between estimated and actual values across varying numbers of regions.

### Challenges of O-PTIR spectroscopy to identify MPs

Generally, for identification of MP using the O-PTIR technique, there are three commonly used methods, i.e., DFIR imaging, point spectra measurements, and HSI. However, each of these methods brings some challenges: for example, DFIR imaging is fast yet provides unreliable results while HSI and point spectra measurements allow for accurate results, but they are time-consuming for data collection. With the QCL system integrated within the O-PTIR microscope, the microscope can generate a single frequency IR image of a 480 μm × 640 μm (spatial resolution: 2 μm) area of a filter in approximately 3 min and 20 s. When an appropriate wavenumber and a threshold value are selected, the generated image shows the majority of MP particles while ruling out most non-MP particles. With this method however, careful selection of a suitable wavenumber and a threshold value for MP particles are necessary; multiple threshold values might be needed in case of interference from the complex non-MP particles. In our study, the discrimination between MPs and non-MP particles based on single-wavenumber images proved to be unfeasible, as illustrated in Fig. S2.

**Figure 1:**
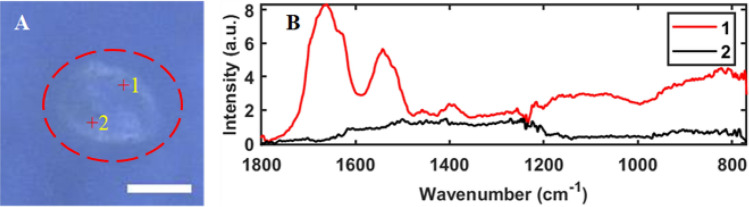
Two spots of the particle encircled in a red dashed line (**A**) selected for point spectra collection and (**B**) the corresponding O-PTIR spectra of the two spots. a.u. is arbitrary units. The scale bar is 20 µm.

The second method commonly used for MP identification is point spectra measurements. After particles are observed in the mIRage microscope, point spectra could be collected for each particle and compared against parent plastic to achieve chemical identification of the particles. This method presented two challenges: (1) When using visible light for particle location under the microscope, non-MP particles were inevitably included as spectral acquisition targets, thus adding to the analysis time. (2) For an individual particle, the O-PTIR spectra could vary significantly across different spots of the particle (see example in Fig. 1). This necessitates the collection of spectra from multiple areas of the particle to enhance the reliability of identification results. Consequently, the analysis time will be multiplied. For example, it takes 25 s to obtain a spectrum (a total of 5 scans acquired for each single spectrum), so if there are 100 particles from the regions of interest on the filter and three spectra are required for each particle, the total analysis time needed is at least 2 h. This estimation only accounts for the raw data acquisition, excluding additional durations associated with manual adjustments such as repositioning the objective or refocusing. In light of this, such an approach becomes exceedingly time-intensive, especially when a vast number of particles are in play.

HSI was the third method employed for MP identification. HSI generates an image where each pixel contains a full spectrum. Hence, it is a reliable method for MP identification. However, this reliability comes at the cost of drastically longer data collection time, which makes HSI impractical for routine MP analysis. For example, capturing a hyperspectral image for a 480 μm × 640 μm area (spatial resolution of 2 μm and spectral resolution of 2 cm^−1^, from a spectral range of 769–1801 cm^−1^) requires almost two weeks.

In response to the challenges mentioned above, we have developed a reliable MP detection framework with an improved speed that is suitable for detecting a large quantity of nylon MPs. It can collect spectral data from nine areas (the size of each area is 480 μm × 640 μm) of a filter (at a spatial resolution of 2 μm) within just approximately 2 h. Powered by machine learning, the reliability of this framework is not compromised in response to reduced data collection time.

### Wavenumber selection and modelling performance

In order to effectively utilize DFIR imaging for high-throughput analysis of MPs, it is crucial to carefully select specific wavenumbers that provide the greatest discriminatory power between MP and non-MP particles. Making incorrect choices in wavenumber selection can directly impact the accuracy of identification. Acquiring too many wavenumbers increases measurement time, resulting in decreased throughput. For instance, adding just one more wavenumber can lead to an approximate 30-min increase in the time required for our proposed MP detection framework to collect data from a single filter. To identify the important wavenumbers and determine the optimal number of such wavenumbers, a database collected from bulk nylon plastic was assembled, containing 1038 spectra of MP and 1052 spectra of non-MP.

**Figure 2:**
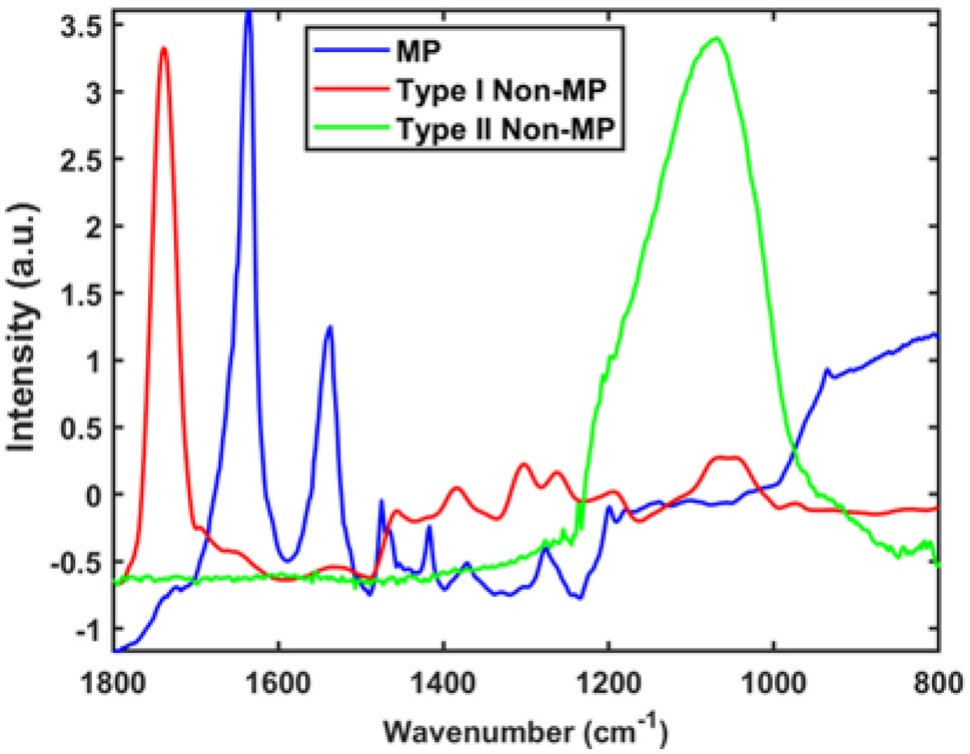
Mean spectra for nylon MP class and two non-MP types from the database constructed, following standard normal variate (SNV) to minimize the multiplicative effects.

We found several types of non-MP particles in our dataset. Figure 2 displays the spectra of two non-MP classes (type I non-MP and type II non-MP), along with the mean spectrum of MP, enabling a comparison. Upon initial inspection, type I non-MP exhibits a prominent sharp peak in the 1700–1800 cm^−1^ spectral range, while type II non-MP displays a broad peak in the 1000–1200 cm^−1^ spectral range. In contrast, the apparent characteristic peaks of MPs are two consecutive sharp peaks in the 1500–1650 cm^−1^ range.

**Figure 3:**
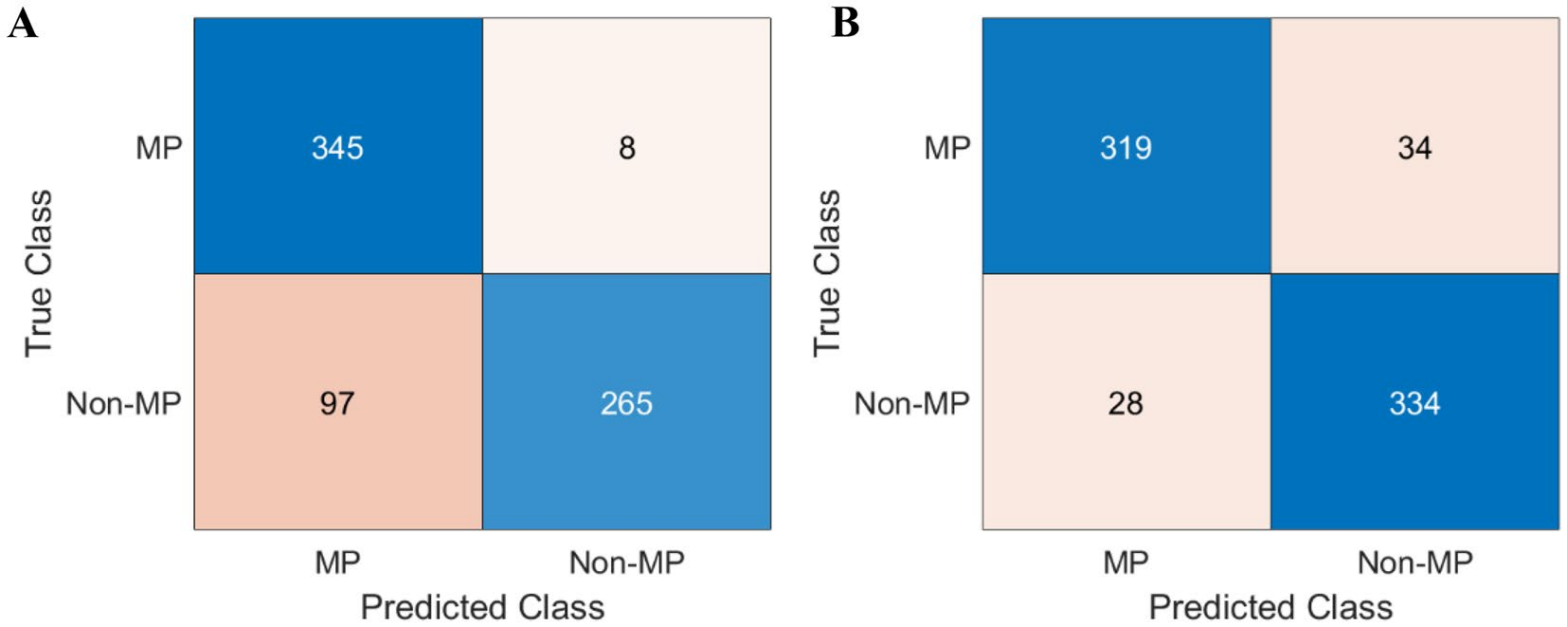
Confusion matrix showing classification accuracy for the test set of SVM-Full model using full spectral variables (**A**) and SVM-Four model (**B**).

Two thirds of the spectra from each class were randomly selected as the training dataset for model development, and the remaining samples formed the test dataset. Based on the obtained results, the model utilizing the full wavenumber spectrum yields a correction accuracy rate of 85.31% (see Table 2). The confusion matrix of the SVM-Full wavenumber model (Fig. 3A) implies that there are 8 point spectra of MPs wrongly classified as non-MPs and 97 of non-MPs mistakenly assigned as MP.

**Table 2:** Performance of SVM (all wavenumbers) and SVM (based on 4 wavenumbers) for the test set in terms of sensitivity, specificity, correct classification rate (CCR) and Matthews Correlation Coefficient (MCC).

	SVM-Full model	SVM-four wavenumbers
Sensitivity	0.9773	0.9037
Specificity	0.7320	0.9227
CCR	85.31%	91.33%
MCC	0.7300	0.8266

**Figure 4:**
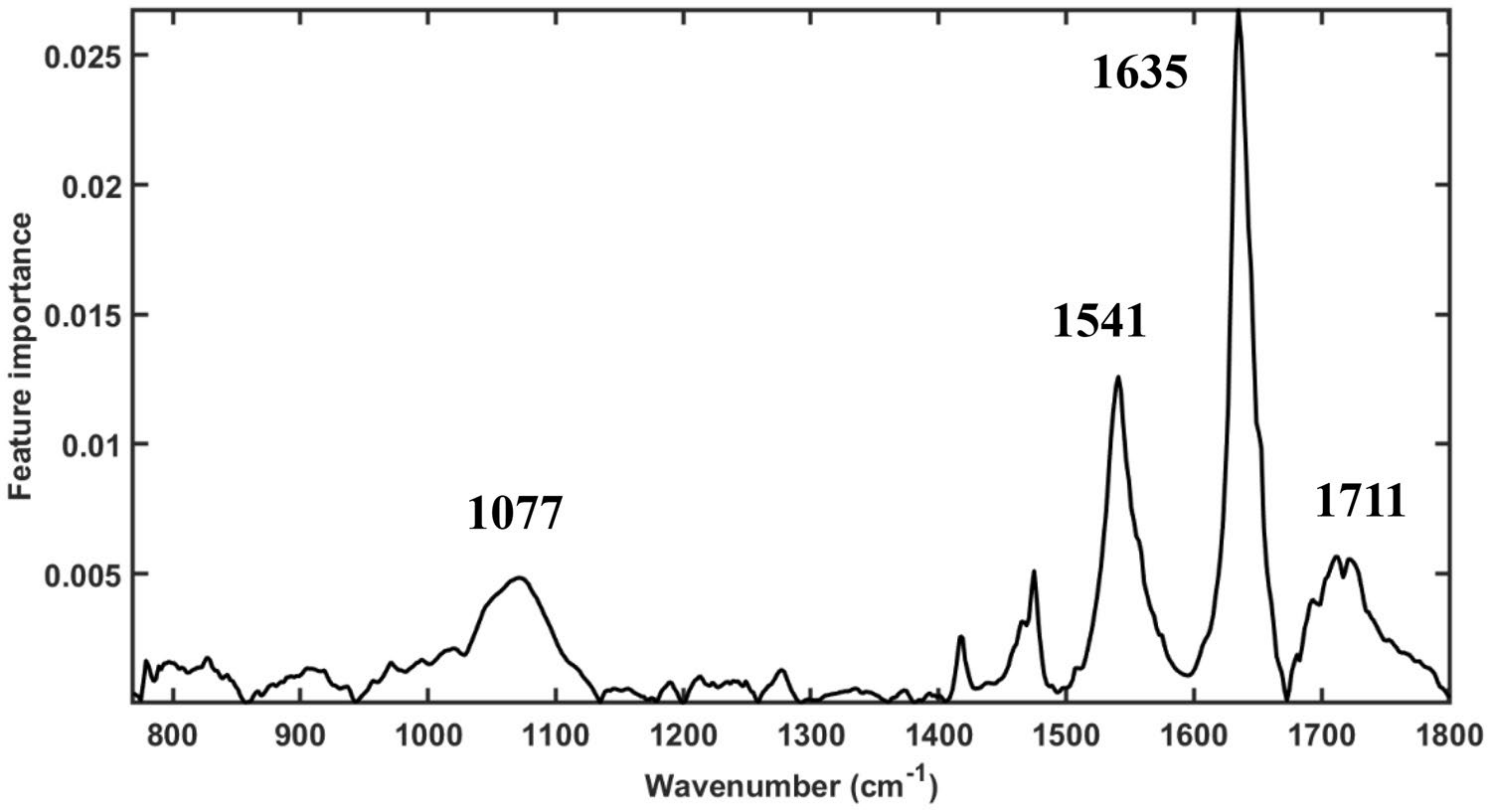
The coefficients (or weights) of the SVM model, which indicate the importance of each feature (wavelength), are then plotted. Four wavenumbers which has relatively higher feature importance than other are marked above the curves (i.e., 1711 cm^−1^, 1635 cm^−1^, 1541 cm^−1^, and 1077 cm^−1^).

Subsequently, the coefficient based feature importance for the full wavenumber model (Fig. 4) was plotted to visualize the contribution of individual spectral variables. According to Fig. 4, we could choose the important wavenumbers to our dataset based on the feature importance. The higher feature importance signifies stronger discriminative capability. Based on the analysis of the coefficients of the SVM-Full wavenumber model, wavenumbers 1711 cm^−1^, 1635 cm^−1^, 1541 cm^−1^, and 1077 cm^−1^ (indicated in Fig. 4) showed the feature importance, hence, were selected as important wavenumbers for distinguishing between MPs and non-MPs. As seen from Table 2, the model optimized with these four wavenumbers demonstrates an enhanced correction rate of 91.33%. Meanwhile, the SVM-Four wavenumbers model (Fig. 3B) resulted in 34 point spectra of MPs wrongly classified as non-MPs and 28 of non-MPs mistakenly assigned as MP, which shows it is a balanced model for classification tasks. The SVM-Four wavenumbers model appears to outperform the SVM-Full wavenumber model in terms of specificity, CCR, and MCC, suggesting that it is a better model for this classification task. However, the SVM-Full wavenumber model has a higher sensitivity, making it better at identifying true positive cases.

**Figure 5:**
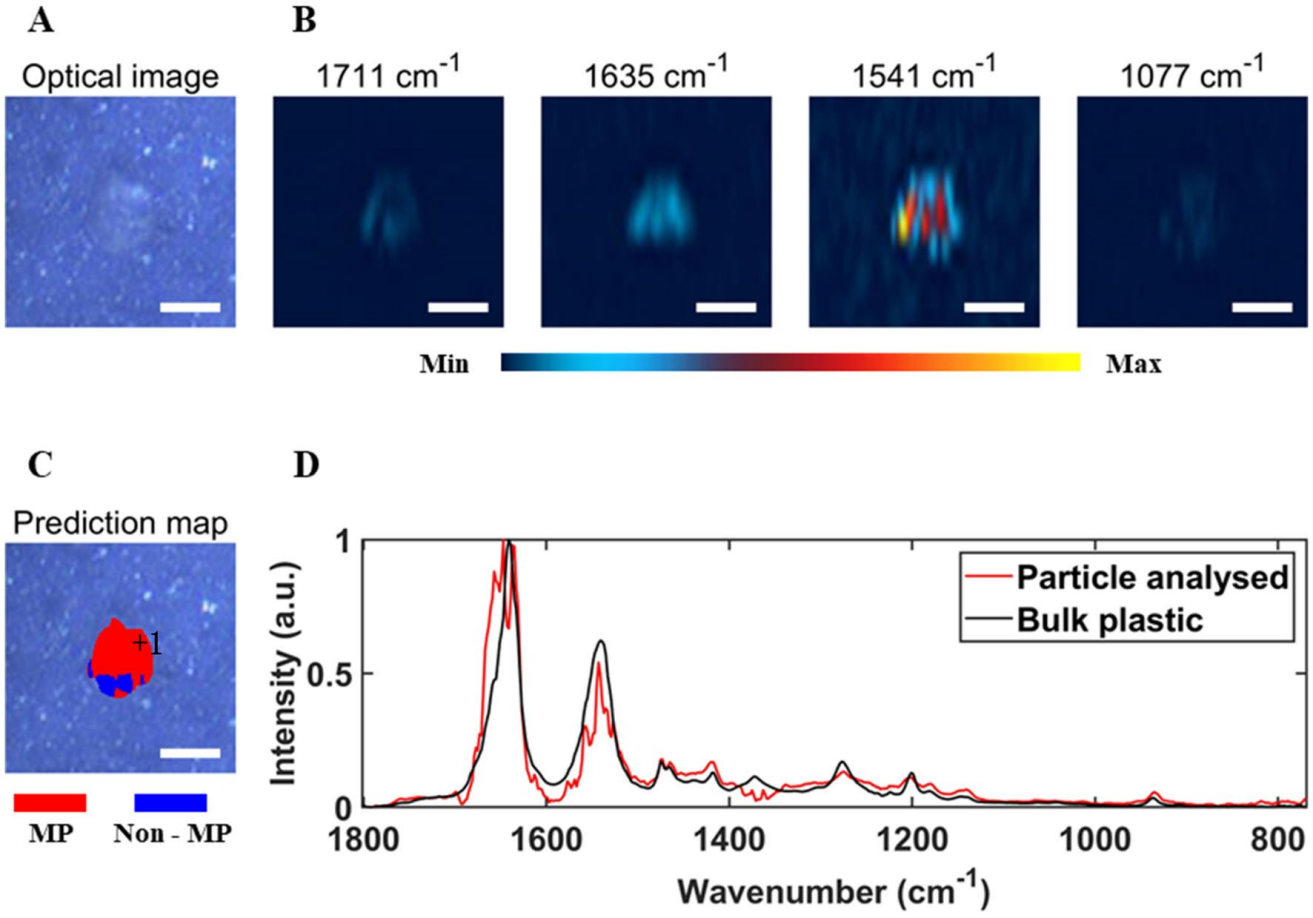
An optical image of an area of a prepared filter, with a MP particle in the center of the image (**A**), single frequency images of that area using 1711 cm^−1^, 1635 cm^−1^, 1541 cm^−1^ and 1077 cm^−1^ band intensity, with the absorbance intensity of each chemical image normalized to the same range (**B**), support vector machine (SVM) prediction results of the particles in this area (**C**), and normalized O-PTIR spectra of the particle and the bulk plastic (**D**). The “ + 1” in (**C**) indicates where the spectrum of the particle in (**D**) was collected. The scale bar is 20 µm.

**Figure 6:**
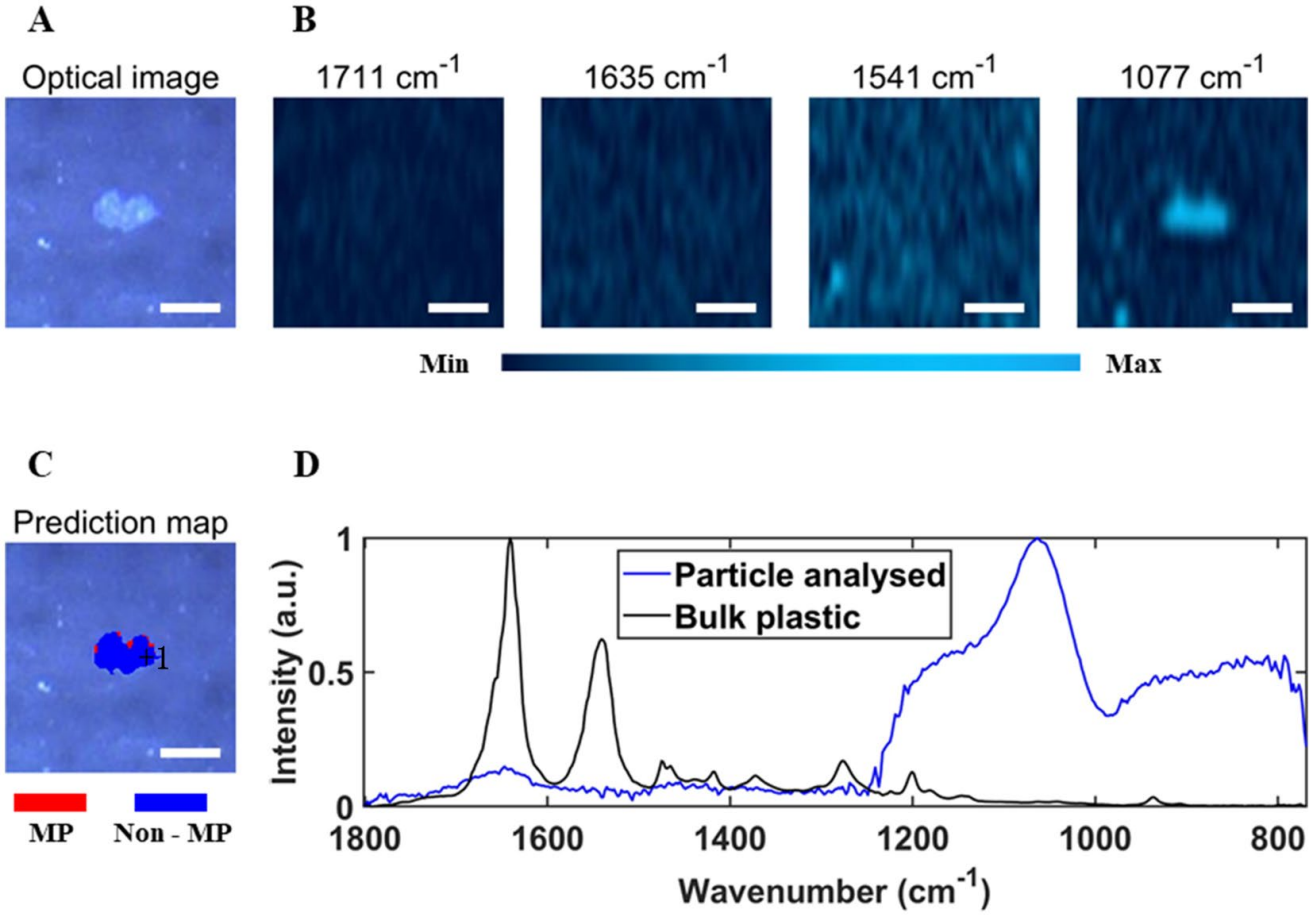
An optical image of an area of a prepared filter, with a non-MP particle in the center of the image (**A**), single frequency images of that area using 1711 cm^−1^, 1635 cm^−1^, 1541 cm^−1^ and 1077 cm^−1^ band intensity, with the absorbance intensity of each chemical image normalized to the same range (**B**), support vector machine (SVM) prediction results of the particles in this area (**C**), and normalized O-PTIR spectra of the particle and the bulk plastic. The “+ 1” in (C) indicates where the spectrum of the particle in (**D**) was collected. The scale bar is 20 µm.

After the selection of the four important wavenumbers, DFIR images were obtained at the important wavenumbers from the nine subsampled regions of the filter. Particle identification could be performed through visual inspection of these DFIR images. For instance, Fig. 5A shows an optical image of a small region of a filter with a particle in the centre, and Fig. 5B shows chemical images of that region based on the intensity of 1711 cm^−1^, 1635 cm^−1^, 1541 cm^−1^, and 1077 cm^−1^ bands. The absorbance intensity of each chemical image was normalized to the same range. The particle in this region exhibits high signal intensity at 1635 cm^−1^ and 1541 cm^−1^, while showing weak signal intensity at 1711 cm^−1^ and 1077 cm^−1^, indicating that it is a MP particle. On the other hand, non-MP particles would show weak signal intensity at 1635 cm^−1^ and 1541 cm^−1^, while showing strong signal intensity at 1711 cm^−1^ and/or 1077 cm^−1^ (See Figs. 6A,B for an example of non-MP particles).

However, for accurate particle identification, visual inspection is not advisable due to low accuracy. Meanwhile, application of SVM-Full model requires a huge amount of time in the collection of point spectra from all particles. Therefore, an SVM-Four wavenumbers model was trained from the four important wavenumbers to predict each particle accurately. Spectral data at the four important wavenumbers were extracted from the same database used for the SVM-Full wavenumber model. The trained SVM model on the selected four wavenumbers demonstrated good performance, evidenced by a high CCR, MCC, sensitivity and specificity (Table 2).

After applying the SVM classifier to the particle in Fig. 5A, each pixel of the particle was labelled as either MP (red) or non-MP (blue), providing an intuitive and accurate identification result. Figure 5C displays the SVM prediction results for one example area. As can be seen, most pixels in the particle have been labelled as MP, with a small portion labelled as non-MP. The result for a particle was determined by the majority vote of the labels of all pixels within the particle. Thereby this particle was identified as a MP particle. This was further confirmed by the full spectrum of this particle (Fig. 5D). Also, by applying the SVM classifier to the particle in Fig. 6A, the particle was predicted to be a non-MP particle (Fig. 6C). Figure 6D presents a spectrum of this particle, which validates the predicted outcome.

Our developed SVM model offers several distinct advantages over the traditional correlation-based method for MP identification. Firstly, the SVM model only requires four wavenumbers as input, significantly reducing the complexity of data collection compared to the correlation-based approach, which involves obtaining spectra from each particle and calculating correlation coefficients. This efficiency translates into a substantial time-saving advantage. Therefore, the developed method is particularly useful when dealing with a large number of particles on the filter. Secondly, the correlation-based method often relies on establishing a threshold for identification, introducing a subjective element into the process. In contrast, the SVM model automates the assignment of particles to MP or non-MP categories, contributing to a more consistent and reliable MP identification process. Last but not least, once essential wavenumbers are identified and a simplified model is developed, the SVM approach can be extended to identify a range of polymers. This versatility is a significant advantage, enabling the model to adapt to various MP compositions beyond the scope of the original correlation-based method.

### Performance of sample processing to reduce non-MPs

Using the novel identification procedure developed, it was possible to investigate the effectiveness of several sample pre-processing steps in a more representative and less biased and efficient way. To this end, high-temperature filtration and alcohol pretreatment were chosen as methods for reducing non-MP. The performance of these two treatments was evaluated separately, including the analysis of the spectra and DFIR images at four selected wavenumbers. The evaluation included an assessment of their impact on the spectra of MP and their effectiveness in removing non-MP. To assess the effectiveness of particle removal, the MP particle/all particle ratio (MP/All) detected by four wavenumbers SVM model was used. A treatment was considered effective if it significantly increased this ratio.

#### Filtration at different temperatures

##### Spectral changes

By boiling the nylon bulk, MP particles were released. The released particles were subsequently enriched on the filters through high-temperature filtration and room-temperature filtration, respectively. The mean spectrum of MP from high-temperature filtration, the mean spectrum of MP from room-temperature filtration, and the mean spectrum of nylon bulk were plotted together for comparison (Fig. S3). Results showed that when the mean spectrum of nylon bulk was compared to the mean spectra of MP (regardless of the filtration temperature), no consistent peak shift was found. When the mean spectrum of MP from high-temperature filtration and the mean spectrum of MP from room-temperature filtration were compared, no consistent peak shift was found either. These findings demonstrate that exposure to high temperatures reaching water boiling point will not impact the spectral profiles of MPs when compared to the original bulk plastic.

##### Effect on reducing non-MP particles

After the thermal degradation of nylon bulk, the particles released were captured on filters through high-temperature filtration and room-temperature filtration, respectively. Using our developed SVM classifier, particles in the nine subsampled regions of the filter were counted and subsequently the ratio MP/All was calculated. The MP/All ratio from the room-temperature filtration was 0.090 ± 0.012, and from the high-temperature filtration was 0.08 ± 0.012, respectively. The normal *t*-test results indicated that the effectiveness of high-temperature filtration in removing non-MP was not evident.

Gerhard et al.^18^ reported that slip agents (such as fatty acid and fatty acid esters) of plastic products are released concomitantly with the release of MP particles, and these slip agents might be dissolved in hot water and washed away during the filtration process. In light of this, our results suggest that the nylon bulk used in our study might have just a small amount fatty acid or their esters. Indeed, Hansen et al.^19^ reported that as additives in plastics, the amount of slip agents could be as low as 0.1%, and the removal of a small amount of additives from MP samples might not statistically significant. Furthermore, based on observations of the prepared filters, we did not see a thin residue on the room-temperature filter, which was observed by Gerhard et al.^18^ who confirmed that most part of the thin residue in their experiment was identified as additives. This supports that the amounts of hot water-rinseable additives in our samples were low, however this would generally be sample specific.

#### Alcohol treatment of filter

##### Spectral changes

After the degradation of nylon bulk in boiling water, the particles released were retained on filters. An alcohol treatment was subsequently applied to the filters to reduce non-MP particles. The mean spectra of MP before and after an alcohol treatment and the mean spectrum of nylon bulk were plotted together and compared (Fig. S4). Results revealed that when the mean spectrum of nylon bulk was compared to the mean spectra of MP (regardless of the alcohol treatment), no consistent peak shift was observed. When the mean spectra of MP before and after the alcohol treatment were compared, no consistent peak shift was observed either.

**Figure 7:**
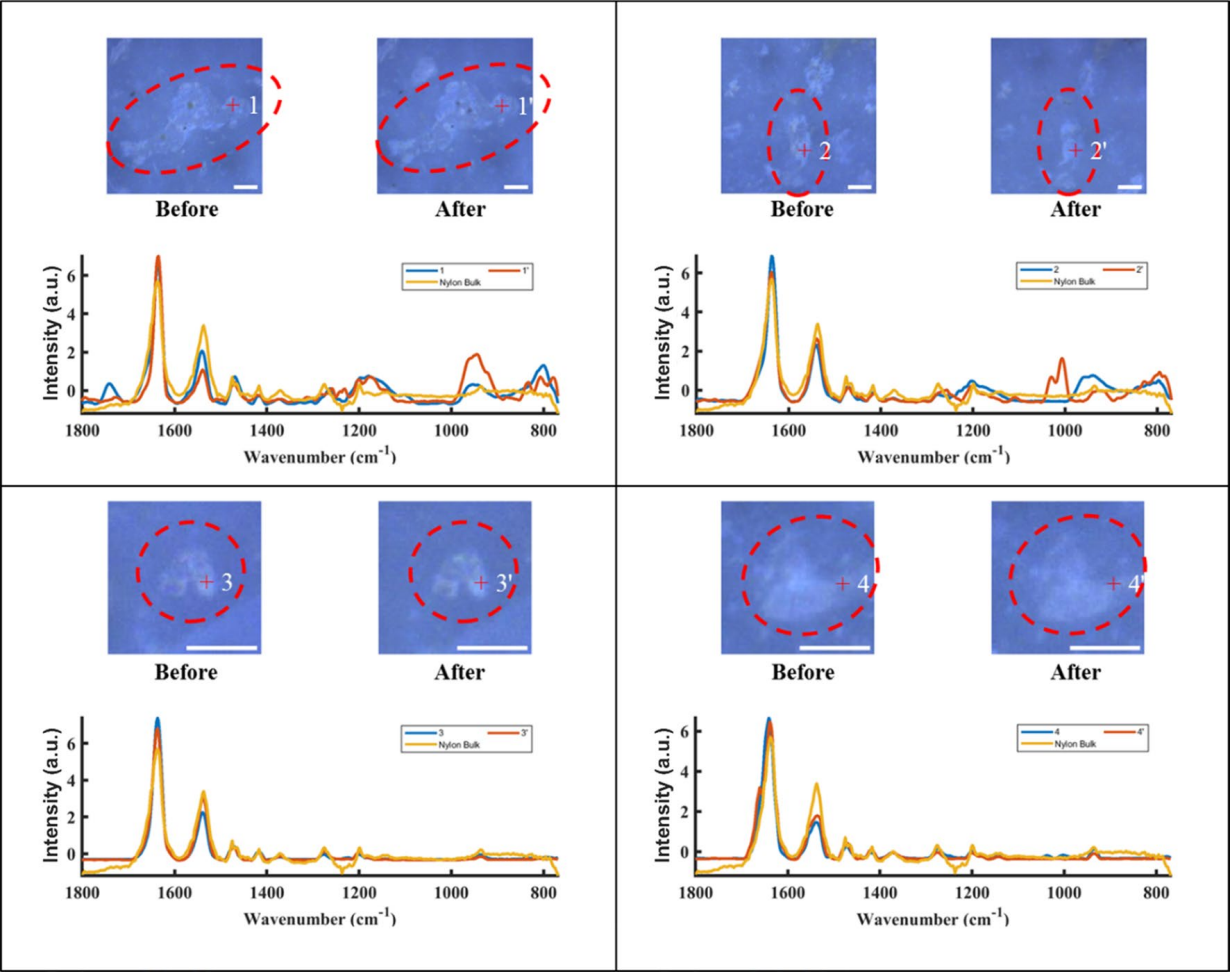
Optical images of nylon MP particles 1, 2, 3, 4, with the particles circled and marked with numbers. The scale bar is 10 µm.

To further explore the effects of alcohol treatment on released particles, this paragraph focuses on spectral changes of individual particles. The spectral data of individual particles was baseline corrected, smoothed, and normalized to between 0 and 1 prior to comparison. Figure 7 shows spectra as well as optical images of 4 MP particles before and after an alcohol rinse. For all four particles presented, peak shifts for signature bands of MP in the range of 769 cm^−1^ to 1801 cm^−1^ were not observed. Particle 1 has a peak at 1741 cm^−1^ before the alcohol treatment; this is a peak that has been assigned to the formation of carbonyl groups during polyamide 66 photo-^20^ and thermal-oxidation^21^, which implicates a pathway of oxidation in hot water for the particles during high temperature treatment (filtration at 70 °C). However, the reduction in signal intensity of this peak after the alcohol treatment might indicate that the alcohol treatment could remove some of the oxidized substances. The spectrum of particle 2 has two new peaks at 1007 cm^−1^ and 1029 cm^−1^, respectively, after exposure to alcohol, which was possibly due to alcohol residue, as these two new peaks correspond to C=O stretching bonds of alcohol^22^. No introduction or disappearance of the peak was observed in the spectra of particle 3 and particle 4. By observing the optical images of these MP particles, it can be concluded that alcohol treatment did not have an effect on their morphology.

##### Effects of reducing non-MP particles

**Figure 8:**
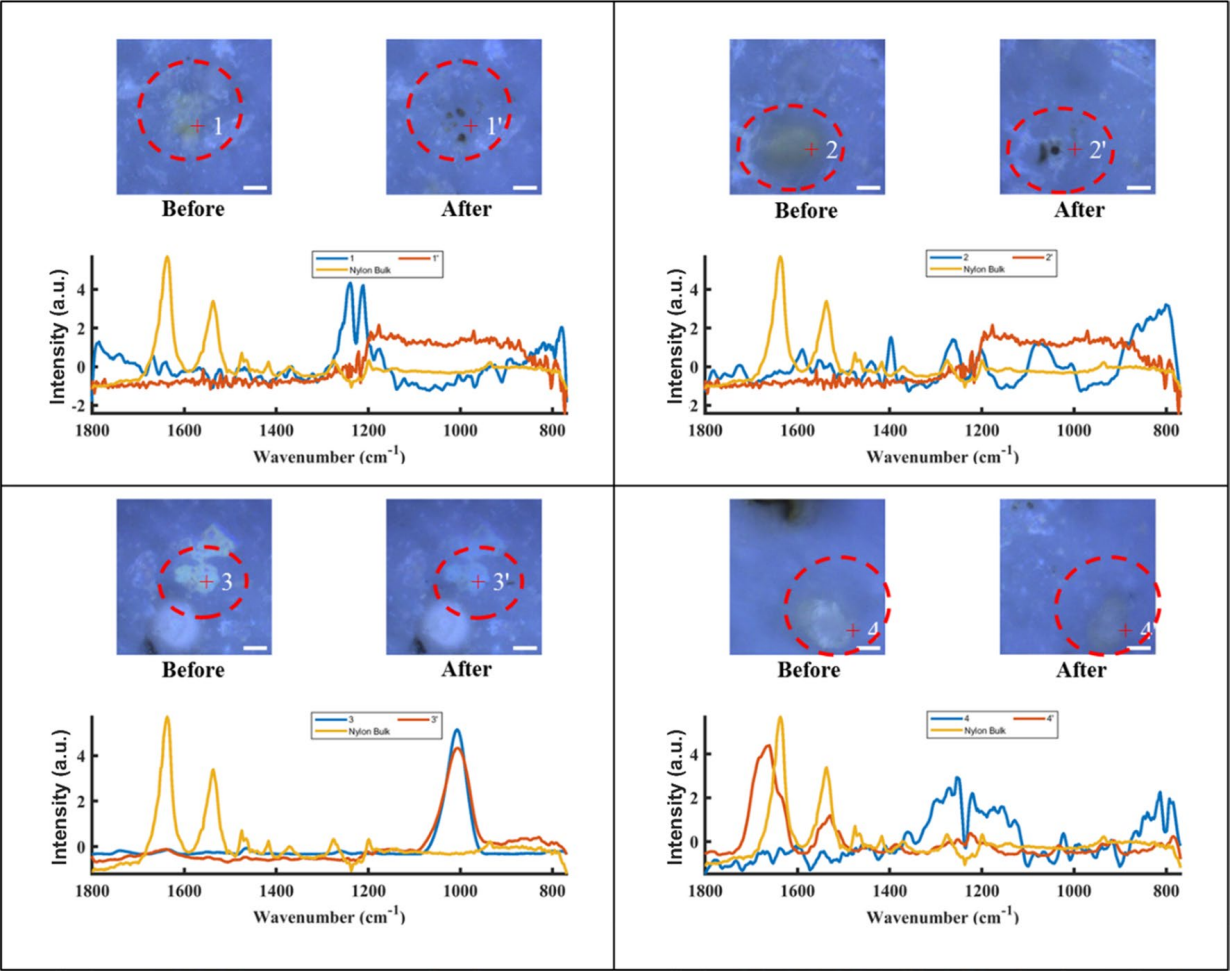
Optical images of non-MP particles 1, 2, 3, 4, with the particles circled and marked with numbers. The scale bar is 10 µm.

Figure 8 shows spectra as well as optical images of 4 non-MP particles before and after an alcohol rinse. Particle 1 and particle 2 appear to be yellowish to brownish. These types of non-MPs are easy to be discriminated against from MPs based on visual observation of optical images, as most of the MP particles in our experiments are whitish, similar to the color of their bulk plastic samples. However, judgement based on color is not always correct. Subsequent spectral analyses confirmed that particle 1 and particle 2 are not MP. After the alcohol treatment, most parts of these two particles were washed away, leaving black remnants on the filter. Though the elimination was not complete, it proved that alcohol could remove non-MP particles. Particle 3 is whitish with a glossy surface, and it is a chlorinated polyethylene particle. After the alcohol treatment, particles with a spectrum similar as chlorinated polyethylene (We do not have any appliances containing polyethylene) remained where it had been, and the spectrum was not changed substantially. The glossiness of the particle was reduced; however, this indicates that alcohol treatment could not remove this type of contaminant. Particle 4 is a white particle, and it is covered by a brown, lumpy object on the upper left. The noise spectrum cannot be identified by the database with high certainty. After the alcohol treatment, it appeared dull grey, and its spectrum looked like that of nylon showing five signature peaks (1633 cm^−1^, 1533 cm^−1^, 1464 cm^−1^, 1416 cm^−1^, 1370 cm^−1^). This implies that the alcohol might be able to remove some contaminants, such as additives, which cover the surface of the MP particle. Li et al.^23^ have reported the same finding that alcohol could wash away some additives attached to the surface of MP particles. The above experiments prove that an alcohol treatment could remove some particle contaminants and wash away some impurities covering MP particles.

To further explore the significance of alcohol treatment, the developed SVM classifier was used to count the particles in the nine subsampled regions of the filter, based on which the MP/All was calculated. The MP/All ratio before the alcohol treatment was 0.129 ± 0.129; and after the alcohol treatment was 0.286 ± 0.207, respectively. The paired *t*-test of the data indicates that an alcohol treatment of the same areas of the filter significantly increases the MP/All (*p* < 0.05). In summary, alcohol treatment was significantly effective in reducing non-MP contaminants.

### Quantification of nylon MPs from teabags

The proposed MP detection framework was specifically adapted for application to detect MPs released from nylon teabags. However, it's important to note that not the entire framework was employed in this context. Rather, a selective application was implemented, excluding the components based on DFIR imaging and the SVM-Four wavenumber model. After steeping teabags in hot water, MPs were released and collected on a filter through filtration at room temperature. This filter was rinsed with alcohol and air-dried in the fume hood prior to O-PTIR data collection. The contaminants from the teabag are not the same as those originating from reference nylon bulk plastics. For example, teabags might have some contaminants from tea residuals, as noted by Xu et al.^13^. Particles released from nylon teabags were identified through point spectra measurements due to the relatively low particle count (i.e., < 5 particles) observed in the subsampled regions of the filter (see “Conventional MP identification”).

**Figure 9:**
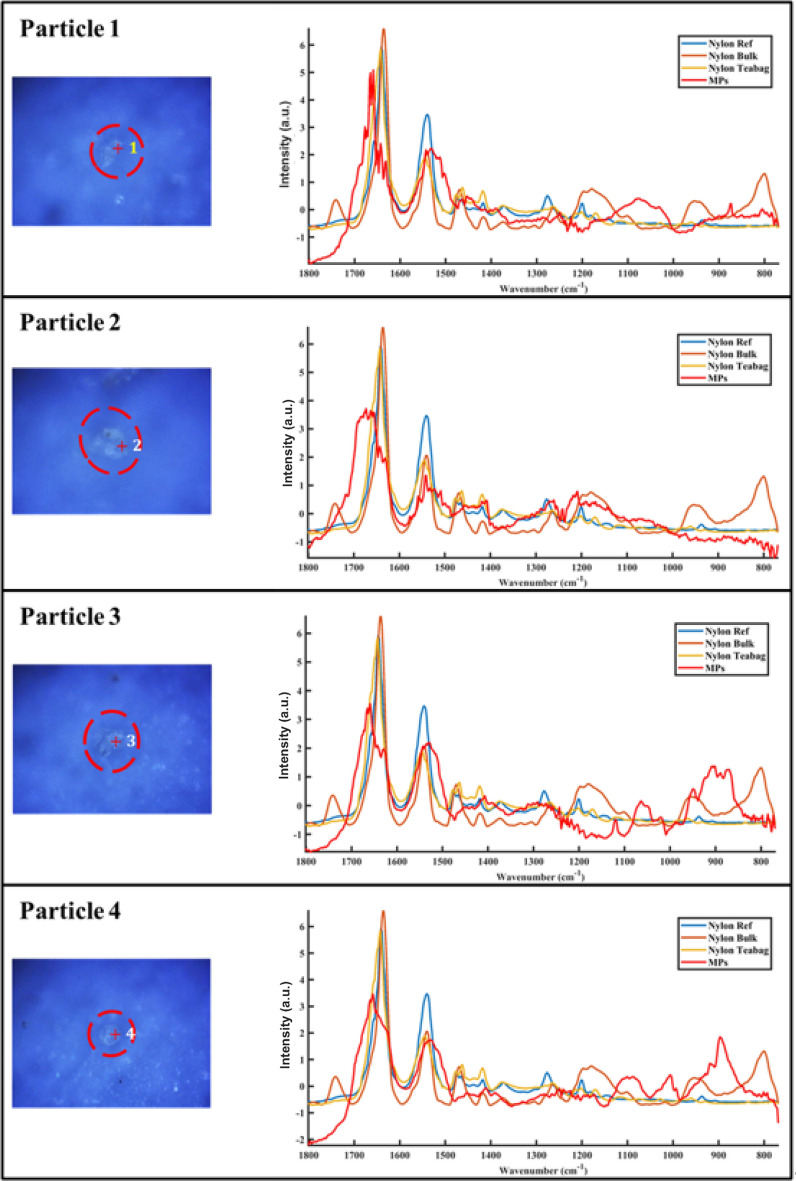
Optical images of nylon MP particles 1, 2, 3, 4 released from nylon teabag, with the particles circled and marked with numbers.

Characterization of MP particles released from teabags was carried out using the MATLAB image processing toolbox function “regionprops”, which calculates properties of each particle including area, length (length of the major axis of the fitted ellipse), width (length of the minor axis of the fitted ellipse), and circularity. In Fig. 9, we present four optical images to show nylon MP particles, which have been released from three nylon teabags; they are circled and marked with numbers. To provide a comprehensive analytical context, the spectra of three key references are plotted alongside: a nylon reference sphere, a sample of nylon in bulk form, and the material of the nylon teabag itself. This juxtaposition allows for a direct comparison between the spectra of the isolated particles and these standard nylon references, this contributes to a more detailed understanding of the appearance as well as the spectral properties of the particles.

**Figure 10:**
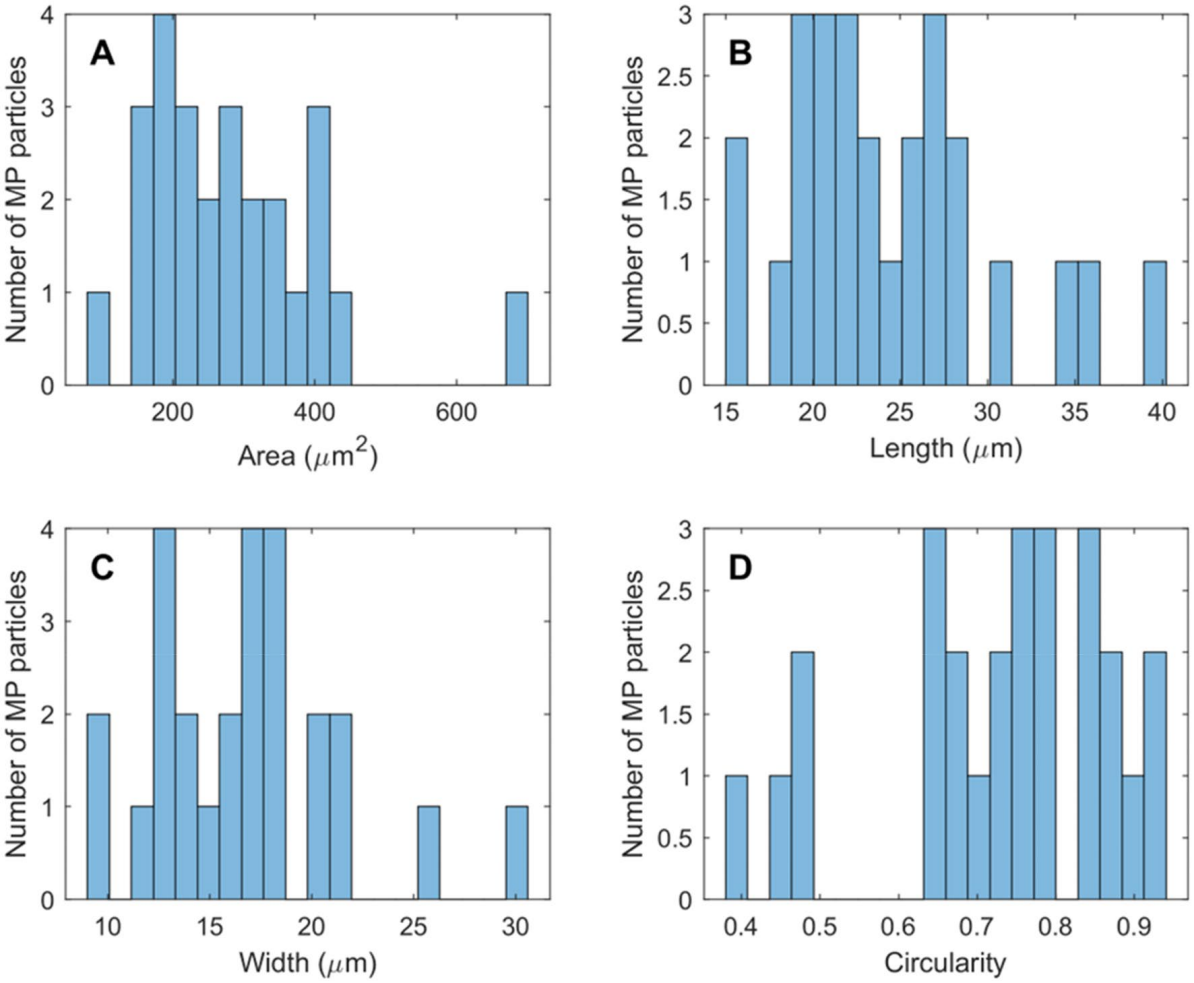
Length (**A**), width (**B**), area (**C**) and circularity (**D**) of MP particles released from steeping a single teabag.

The average quantity of MP in the nine subsampled regions of the filter was 8.7 ± 1.2. Extrapolating to the whole filter, we would estimate 319 ± 43.7 MP particles released from steeping three teabags, or approximately 106.3 ± 14.6 MP particles were released from one teabag. The particle counts/quantities of MPs released from teabags previously reported are listed in Table 1. Our reported count is comparable to that reported by Ouyang et al.^9^, who found 393 MPs using FTIR-based particle-based analysis, although their brewing time was much longer than ours (1 h vs 5 min). Regarding Hernandez et al.^7^, their brewing temperature and time are very similar to ours. Nevertheless, as they did not conduct particle-based analysis, their results were overestimated^8^. The detection limits of O-PTIR spectroscopy and Raman spectroscopy are similar, with O-PTIR spectroscopy being around 500 nm and Raman spectroscopy being around 1 µm. Based on this, we were surprised to find that the number of MPs we detected was one to two orders of magnitude lower than the 5,800 – 20,400 per teabag (brewed at 95 °C for 5 min) reported by Busse et al.^8^ using Raman spectroscopy. Busse et al.^8^ conducted particle-based analysis, indicating that their results should be considered reliable. However, it is important to note that their use of Raman spectroscopy may have led to misidentification of non-MP particles as MPs in an unexpected way. To illustrate, Busse et al.^8^ identified and counted polyethylene (PE) particles in the teabag leachate. However, these PE particles could also be behenamide (CH_3_(CH_2_)_20_CONH_2_), which is a typical slip additive widely used in PE plastic. Behenamide exhibits a high level of spectral similarity with PE in Raman spectroscopy, up to 90%, mainly due to the strong Raman signal associated with its saturated alkyl chains (i.e., ν(C–H)) and relatively weak Raman signals from carbonyl and amine groups^23^. The observed disparities between our results and those of Busse et al.^8^ could also potentially be attributed to the use of different types of teabags. The counts/quantities reported by other studies listed are expressed in the mass of MPs released per teabag^11,12^, or the number of MP particles per kg of teabags^10^. Therefore, direct comparisons with these studies are not possible in our paper. Subsequently, the length, width, area, and circularity of each particle were measured and calculated using the MATLAB function “regionprops”. Figure 10A shows the surface area of the MP particles. Except for the two MP particles with the smallest (100 µm^2^) and largest (680 µm^2^) surface area, the majority of the remaining particles have surface areas ranging from 150 to 550 µm^2^. Figure 10B shows the distribution of the length of MP particles. As can be seen, the maximum length is 40 µm and the minimum length is 16 µm, while most MP particles have a length ranging from 18 to 28 µm. Figure 10C displays the width of the MP particles. As seen from the graph, the smallest width is 9 µm, while the largest width is 30 µm. The majority of the MPs have a width range between 12 and 24 µm. Figure 10D shows the circularity of the MP particles. Among all the MP particles, only 4 have a low circularity (0.1–0.4), while most of the MP particles have circularity ranging from 0.65 to 0.95. Circularity is a measure of how closely a shape resembles a perfect circle. Circularity values near 1 represent perfect circles, while values close to 0 indicate shapes that deviate significantly from circularity. Based on the literature, particles that are more circular in shape are found to be less toxic, while those that deviate from a circular shape, manifesting more stretched or fiber-like, are associated with a higher level of toxicity^24^.

## Method and materials

### Precautions for contamination prevention

During the experiment, strict protocols in the lab were followed to ensure the reliability and accuracy of the results obtained. Clean glass containers were used; cotton-based lab coats and nitrile gloves were worn consistently throughout the experiment; all sample preparation procedures were carried out within a fume hood (WS(G)-71 1800 mm STO VAV); prepared samples were stored in glass petri dishes with lids covered, except during the sample scanning. Finally, each experiment was carried out in triplicate using the same established protocol to ensure that the results obtained were repeatable and of a high standard.

Anodisc aluminum oxide filters (diameter: 25 mm, 0.2-micron pore size, herein referred to as “the filter”) were used to collect MPs. To monitor the occurrence of contamination during each experiment, a negative control was also analysed, using a blank filter subjected to the same processing steps alongside the actual samples. The detection of particles on the blank filter was carried out under the brightfield microscope of the OPTIR system, by visual inspection. It was used to monitor airborne contaminants, as well as potential contaminants originating from the deionized (DI) water, reagents, laboratory surfaces, equipment, and the clothing and gloves worn by the operator.

### Sample preparation

#### Preparation of MPs from bulk nylon

To mimic the real-life scenario of MPs released from plastic products and investigate the effectiveness of alcohol treatment and high-temperature filtration in removing non-MP particles, we utilized 12 pieces of bulk nylon 6/6 samples (CS HYDE Company Product: 31-30F) with a thickness of 1.604 mm, each measuring 1.5 cm in length and width. In order for MPs to be released, the bulk samples were boiled in DI water for four minutes on a hot stirrer (Stuart UC152) without using the magnetic stirrer. The nylon plastic pieces were then removed from the leachate, which was left to cool to room temperature (approximately 24 °C, a clean metal thermometer was used to monitor the temperature). Subsequently, the leachate was poured into a filtration apparatus (Clamped Vacuum Filter Holders, EMD Millipore Corporation). The filter was applied to collect particles from the leachate.

#### Preparation of MPs from teabags

Niks teabags were purchased from a local supermarket (Tesco Extra, Finglas, Dublin, Ireland) (Tea Brand: Niks, Production place: Dublin, Ireland, Best before Date: 02/21, Batch Number: 256727, Tea type: Lemon and Ginger Herbal Tea) and MP collection procedure was conducted according to Xu et al.^13^. In brief, three teabags were included for each replicate, and tea leaves were carefully removed by steel tweezers and thoroughly rinsed with DI water to eliminate any visible tea residue. The rinsed teabags were then dried in the fume hood. Subsequently, following a procedure resembling tea brewing in real-life scenarios, these three teabags were immersed in a beaker containing 50 mL of boiling DI water (approximately 100 °C) without further heating. After a 5-min steeping period, the teabags were removed from the beaker, and the leachate in the beaker was allowed to cool to room temperature, followed by the filtration step.

### Filter holder and filter subsampling

In order to carry out O-PTIR measurements of the Anodisc aluminum oxide filters, we designed a sample holder with a hole in the center, as shown in Fig. S5. The sample holder was printed using a 3D printer (UltiMaker Polylactic acid Silver metallic 9088 plastic, ULTIMAKER 3 supplied by UltiMaker). The diameter of the hole was approximately equivalent to that of the filter used in our experiment. To avoid movement during spectral collection, we applied Blu tack at the edge ring of the filter that is a 2.5 mm outer ring of polypropylene, ensuring the same region of interest (ROI) was localized before and after subsequent alcohol treatment to remove non-MP particles (described further below).

In this experiment, a subsampling strategy was employed when analyzing the filters, and the results were extrapolated to the entire filter. This approach is commonly used when analyzing filters through microscopy, as analyzing the entire filter can be highly time-consuming. Nine evenly distributed areas, each measuring 480 μm × 640 μm, were selected as the subsampled regions on the filter (refer to Fig. S6 for a map and coordinates of the subsampled regions). Collectively, these subsampled regions account for 0.87% of the total filter area. The coordinates of these nine regions could be saved in the local PC (the detailed coordinates are shown in Fig. S6) and combined with the sample holder we designed to avoid operator bias due to manual selection.

The representativeness of these subsampled regions was confirmed through the following steps. Firstly, 0.05 mg of standard nylon microspheres (density: 1.13 g/cm^3^, average diameter: 15–20 μm, in powder form, obtained from Sigma-Aldrich Company Ltd.) were dispersed in 100 ml of deionized water and subsequently captured using the filtration apparatus and the filter. Secondly, based on the supplier's information which assumes that the particles could be treated as spheres, the total mass of particles in these subsampled regions was calculated. This calculation utilized the particle count (acquired by counting the number of particles in the subsampled areas, refer to Fig. S7 for an example), average diameter, and density. Through data extrapolation, the total mass of particles on the entire filter was estimated. In the methodology under consideration, the quantification of nylon spheres relies on spectral analysis. Specifically, particles are identified as nylon-based if their spectral characteristics closely align with a predefined standard nylon spectral profile. Additionally, the algorithm excludes particles that exhibit a rounded morphology but do not exceed a certain threshold in the Z-coordinate relative to the filter plane. This ensures a more accurate and selective count of nylon spheres. Finally, the representativeness of the nine subsampled regions was determined by the ratio of the estimated mass of particles on the filter to the initial 0.05 mg.

### Sample treatments to remove non-MP particles

#### High temperature filtration

Some plastic additives can dissolve in hot water, thereby their precipitation on the filter during filtration could be prevented by a high temperature filtration^18^. The preparation of MP samples released from bulk nylon involved two distinct conditions: room temperature filtration, which entailed cooling the leachate to room temperature prior to filtration, and high temperature filtration, which involved cooling the leachate to 70 °C instead of room temperature. Three procedural blanks were prepared in accordance with the same experimental conditions (room temperature, high temperature, and teabags steeping into the hot water) as the samples. These blanks serve as control replicates designed to assess potential contamination from the environment.

#### Alcohol treatment

Li et al.^23^ recently reported that alcohol treatment of the filter can effectively remove some microplastic additives that interfere with MP identification, including additives adhered to the surface of MP particles. For exploring the effects of alcohol on MPs and non-MPs. An alcohol rinse was implemented after filtration. Around 30 ml of room temperature alcohol (LENNOX Isopropyl Alcohol 70% filtered) was used to rinse the prepared filters. During the rinse, the filter holder was oriented at a forty-five-degree angle to facilitate the even distribution of alcohol across its surface. A pipette gun was utilized to transfer alcohol. Alcohol treatment was applied to filters prepared from both nylon bulk and teabags.

### Identification of MP

The identification of MPs in this study was achieved using the O-PTIR technique. The O-PTIR technique can be elucidated as a "pump-probe" architecture, where the QCL IR laser (the pump) is used to excite the sample; IR absorption of the sample causes local thermal expansion (of the sample), which is then probed using a 532 nm visible laser beam^17^. O-PTIR employs a continuous-wave visible-laser probe to detect the photothermal response of mid-IR-absorbing regions in a sample, eliminating the need for sample contact. This pump-probe mechanism enhances both spatial resolution (approximately 400 nm) and sensitivity (approximately 0.4 pg) when compared to Fourier Transform Infrared Spectroscopy (FTIR)^25^. O-PTIR spectra exhibit a high degree of consistency with FTIR spectra, with a similarity of approximately 99%^25^. As a result, O-PTIR spectra can be readily compared with the FTIR spectral database.

The O-PTIR microscope used was a mIRage IR microscope (Photothermal Spectroscopy Corp, Santa Barbara, CA, USA) integrated with a four-module-pulsed quantum cascade laser (QCL) system, with a tunable spectral range from 1801 to 769 cm^–1^. Prior to data collection, the wavenumber was calibrated at 1641 cm^−1^, a setting substantiated by existing literature on the infrared spectral characteristics of nylon standards. Repeated opto-thermal photothermal infrared (O-PTIR) measurements of nylon bulk (n = 12) validated this calibration, with five measurements manifesting a peak at 1641 cm^−1^ and seven at 1639 cm^−1^. This observed spectral variance falls within the ± 0.2% variability reported by^25^, thereby justifying the acceptability of calibrating the wavenumber at either 1639 cm^−1^ or 1641 cm^−1^. Optical images were acquired using the low-magnification 10× objective and the high-magnification 40×, 0.78 NA objective. O-PTIR spectra were collected in reflectance mode with a step of 2 cm^−1^ through the high-magnification objective. A total of 5 scans were acquired for each single spectrum. Single frequency images were collected at predetermined wavenumbers using the high magnification objective as well.

#### Conventional MP identification

In the case where only a few particles (less than 5 particles in one region) were in a subsampled region of the filter, the Pearson correlation coefficient (based on the 4 selected wavenumber from SVM full wavenumber model) was employed to identify MP particles separately in MATLAB. For each particle in these nine subsampled regions, three spectra of different areas of the particle were collected. These three spectra were then pre-processed and independently compared to the spectrum of the parent plastic (i.e. nylon) using the Pearson correlation coefficient, and once any of the three comparison results was greater than 0.7 (i.e., Pearson correlation coefficient > 0.7), the particle was identified as a MP particle; when the comparison result was between 0.6 and 0.7, the identification was carried out manually based on the spectral peaks. If the result was smaller than 0.6, the particle was identified as a non-MP particle, in our experiment it was identified as a non-nylon MP.

The spectral preprocessing, including Savitzky-Golay smoothing (polynomial order 3, side points 7), baseline removal (strength 17), and normalization, was performed using the PTIR Studio software (Version 4.4.8208, Photothermal Spectroscopy Corp, Santa Barbara, CA, USA, https://www.photothermal.com/). The Pearson correlation analysis was conducted in MATLAB (version 2022b, The MathWorks Inc., Natick, MA, USA, https://www.mathworks.com/) after the spectral preprocessing steps.

#### Developing a discriminant model for MP identification

**Figure 11:**
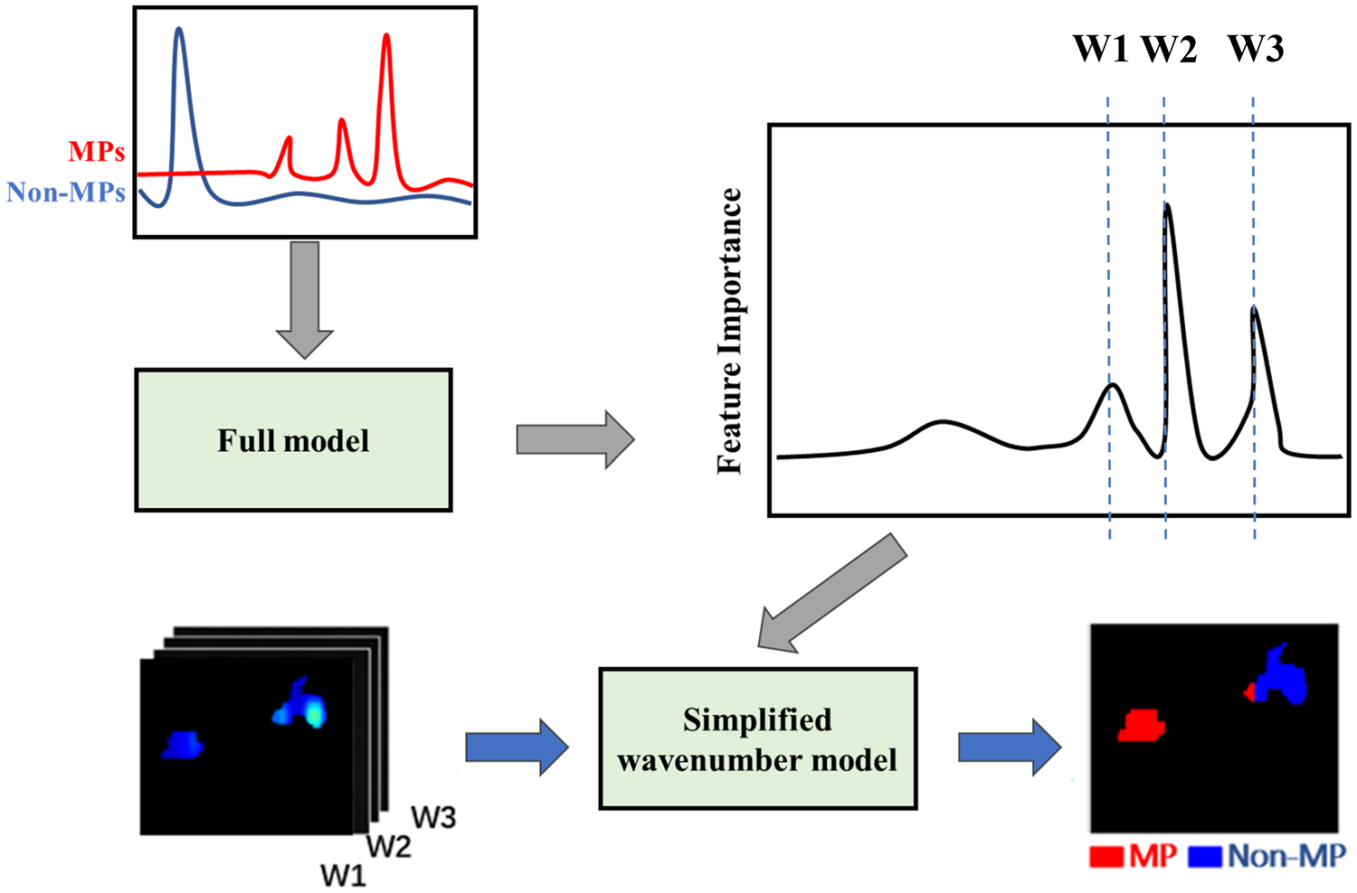
Flowchart of the development and application of the innovative MP detection framework. The grey arrows represent the process of model development, while the blue arrows represent the process of model application.

In situations where a large number of particles (> 5) are present in the subsampled regions, collecting point spectra and using the Pearson correlation coefficient to identify MP became impractical, as data collection time increased tremendously. Hence, an innovative MP detection method based on DFIR imaging and machine learning was proposed. As can be seen from Fig. 11, the detection method started with the collection of MP and non-MP spectra from the particles released from nylon bulk. This collection process is carried out by selecting particles in 9 spatial regions on the filter; spectra similar to the nylon standard are placed in the MP dataset, where ‘similar’ is defined as having the same or similar wavenumber as the main peaks appearing in the nylon standard, and vice versa in the non-MP dataset. (Note: from this point onwards until the end of this paper, "MP" refers to nylon MP, and "non-MP" refers to substances other than nylon MP.). Standard normal variate (SNV) was used to pre-treat spectra prior to model development. A full wavenumber SVM model was subsequently developed to discriminate between MPs and non-MPs and identify important wavenumbers. Based on this, several important wavenumbers could be determined. Using spectral data at these selected wavenumbers (W1, W2, W3 in Fig. 11), an SVM-Four wavenumbers model was developed. The performance of both kinds of SVM models (i.e., full wavenumber v’s 4 selected wavenumbers) was evaluated by computing their correct classification rate (CCR), Matthews correlation coefficient (MCC), sensitivity, and specificity on the test set.

For the application scenario, optical images as well as single frequency images at the selected wavenumbers were first collected at nine regions. Then a mask removing the background was manually created based on the optical image. Finally, the mask and the single frequency images were inputted into the pre-trained SVM model for classification, where each pixel within the mask was labelled as either MP or non-MP. The result for a particle was determined by the majority vote of the labels of all pixels within the particle.

The development, application of the full wavenumber and 4 wavenumber SVM models and masking (i.e., selecting ROIs) were all conducted using MATLAB (Including Image processing & Machine Learning Toolbox).

## Conclusion

In conclusion, this study has successfully developed a robust and reliable method for identifying and quantifying nylon MPs released from nylon bulk plastic and nylon teabags using O-PTIR spectroscopy. The optimized sample preparation process and experimental design demonstrated the potential of O-PTIR as an effective tool for analyzing MPs released from real-life nylon products.

We established the MP characterization methods based on a repeatable method with a 91.33% correct classification rate. For scanning and analyzing an area at low magnification, the proposed method takes about 2 h to collect O-PTIR images at four important wavenumbers to classify all particles by SVM-Four wavenumbers model, which is more efficient than applying HSI.

Based on the results of our teabag experiments, our method can significantly reduce the time required for this process while maintaining the efficient identification of MPs. Concurrently, it allows for the calculation of the length, width, size, and circularity of the identified MPs. Building upon relevant literature, we can then evaluate the potential hazard these released particles may pose to human health. Future research should primarily focus on refining the current method and validating its applicability across a broader range of plastic products. This implies improving the system including but not limited to data augmentation, rigorous validation across diverse products, and algorithmic refinements for enhanced accuracy, efficacy, and inclusivity to potentially encompass a more diverse variety of products. Such improvements can help ensure that our approach to MP detection and measurement is not limited to a narrow category of items, thereby increasing its utility in practical scenarios. The development of this method is essential to address the growing concerns of MP contamination and its impact on the environment and human health.

### Supplementary Information

Supplementary Information.

## Data Availability

The datasets used and/or analysed during the current study available from the corresponding author on reasonable request.
