# Supplementary material for: Machine learning driven methodology for enhanced nylon microplastic detection and characterization
Source: Sci Rep. 2024 Feb 12;14:3464. doi: 10.1038/s41598-024-54003-1 (PMC10859384; doi:10.1038/s41598-024-54003-1)
Supplement: Supplementary file 1 — Supplementary Information. [file 41598_2024_54003_MOESM1_ESM.docx]

# **Advancing Accurate Nylon Microplastic Detection and Characterization: Methodology Development based on Optical Photothermal Infrared Spectroscopy**

**^+^Cihang Yang, ^+^Junhao Xie, Aoife Gowen**^*^**and Jun-Li Xu^*^**

*School of Biosystems and Food Engineering, University College Dublin, Belfield, Dublin 4, Ireland*

^*^ Corresponding author: aoife.gowen@ucd.ie, [junli.xu@ucd.ie](mailto:junli.xu@ucd.ie)

^+^ Co-First Authors: Cihang Yang and Junhao Xie contributed equally to this work.

**
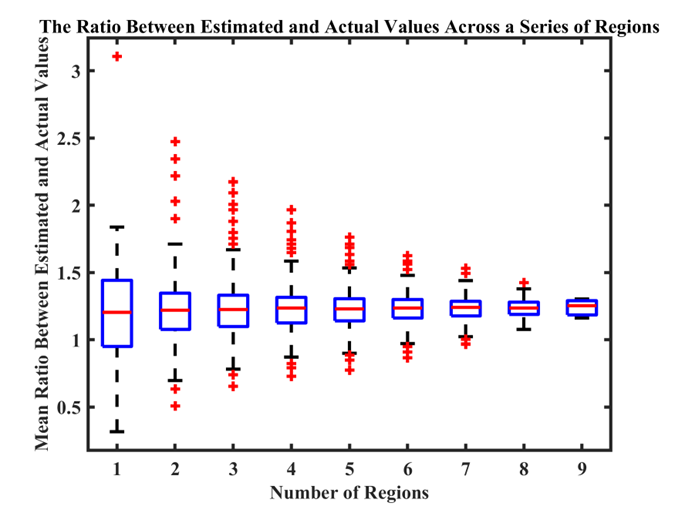
**

**Fig.S1.** The mean value of Ratio Between Estimated and Actual Values Across a Series of Regions in all possible combinations

**Fig. S1** illustrates this relationship using a box plot, where each box) represents the distribution of estimated values from the number of regions selected in all possible combinations (the same regions are selected for all three replicates, for example, if the first region is selected for Rep1, then the same first region is selected for the second and third regions). In conclusion, with the number of regions increases, the variability in the ratio between estimated and actual values tends to decrease, which means that the estimated values are closer to the mean values. This also shows that the risk of under or oversampling reduces as the number of regions is increased.


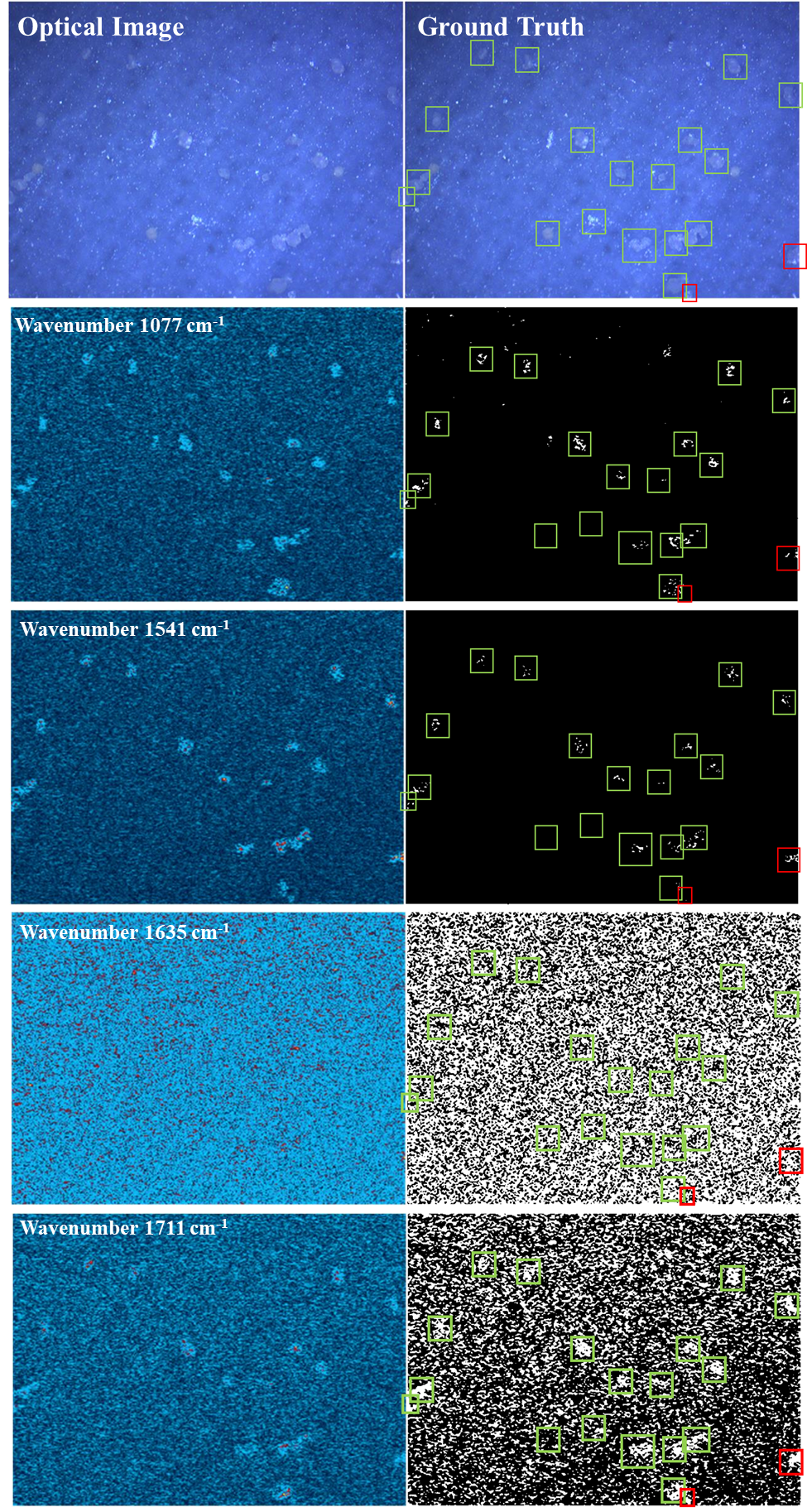


**Fig. S2.** An optical image serves as the ground truth, in which particles identified as 'MP' (Micro-Plastics) are outlined in green boxes, and those labeled as 'non-MP' are outlined in red boxes. Corresponding OPTIR (Optical Photothermal Infrared) images are generated for four specifically chosen wavenumbers. Otsu's method is utilized to establish the optimal threshold for each wavenumber. Subsequently, this threshold is applied to mask the OPTIR images, yielding clearly differentiated results. To facilitate a direct comparison with the ground truth, the results are also demarcated with green and red boxes.

In this figure, we present O-PTIR images captured at four selected wavenumbers from a specific region of interest which contains both MPs and non-MPs. We subsequently applied Otsu’s method to these images in an attempt to choose an ideal threshold for differentiating between MPs, non-MPs, and background. However, our results indicate that relying on images from any single wavenumber is insufficient for setting a definitive threshold to distinguish between MPs and non-MPs, as non-MPs and MPs are indistinguishable in the single wavenumber images. The ground truth of the Optical image was established by collecting spectra for all particles in this image. Since this image is derived from a filtered sample of nylon reference spheres, the nylon particles have a spectral shape closer to the standard nylon spectrum, which also facilitates the establishment of the ground truth.

**Fig. S3.** Mean Spectrum (R1_Room Temperature: 140, R1_High Temperature: 319; R2_Room Temperature: 120, R2_High Temperature: 161; R3_Room Temperature: 73, R3_High Temperature: 115) of three replicates of samples prepared on the same day by different treatments. Comparison of Nylon Reference Sphere spectra after High Temperature and Room Temperature treatments as well as the Nylon Bulk spectra.

**Fig. S4.** Mean Spectrum (R1_Before Alcohol: 140, R1_After Alcohol: 59; R1_Before Alcohol: 120, R1_After Alcohol: 64; R1_Before Alcohol: 50, R1_After Alcohol: 50) of three replicates of samples prepared on the same day by different treatments. Comparison of Nylon Reference Sphere spectra with and without alcohol treatments as well as the Nylon Bulk spectra.


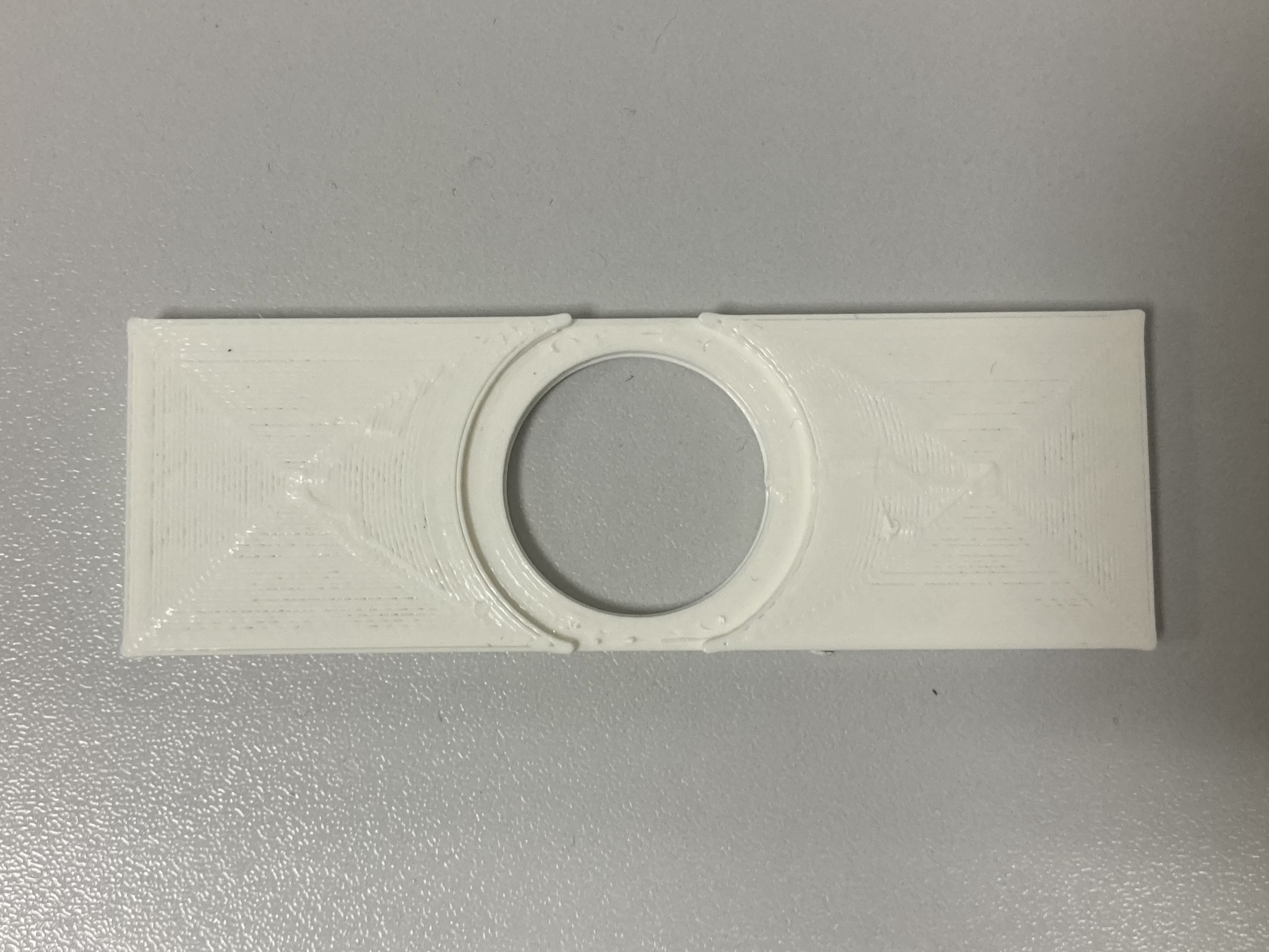


**Fig. S5.** The 3D printed substrate with a hole in the middle.


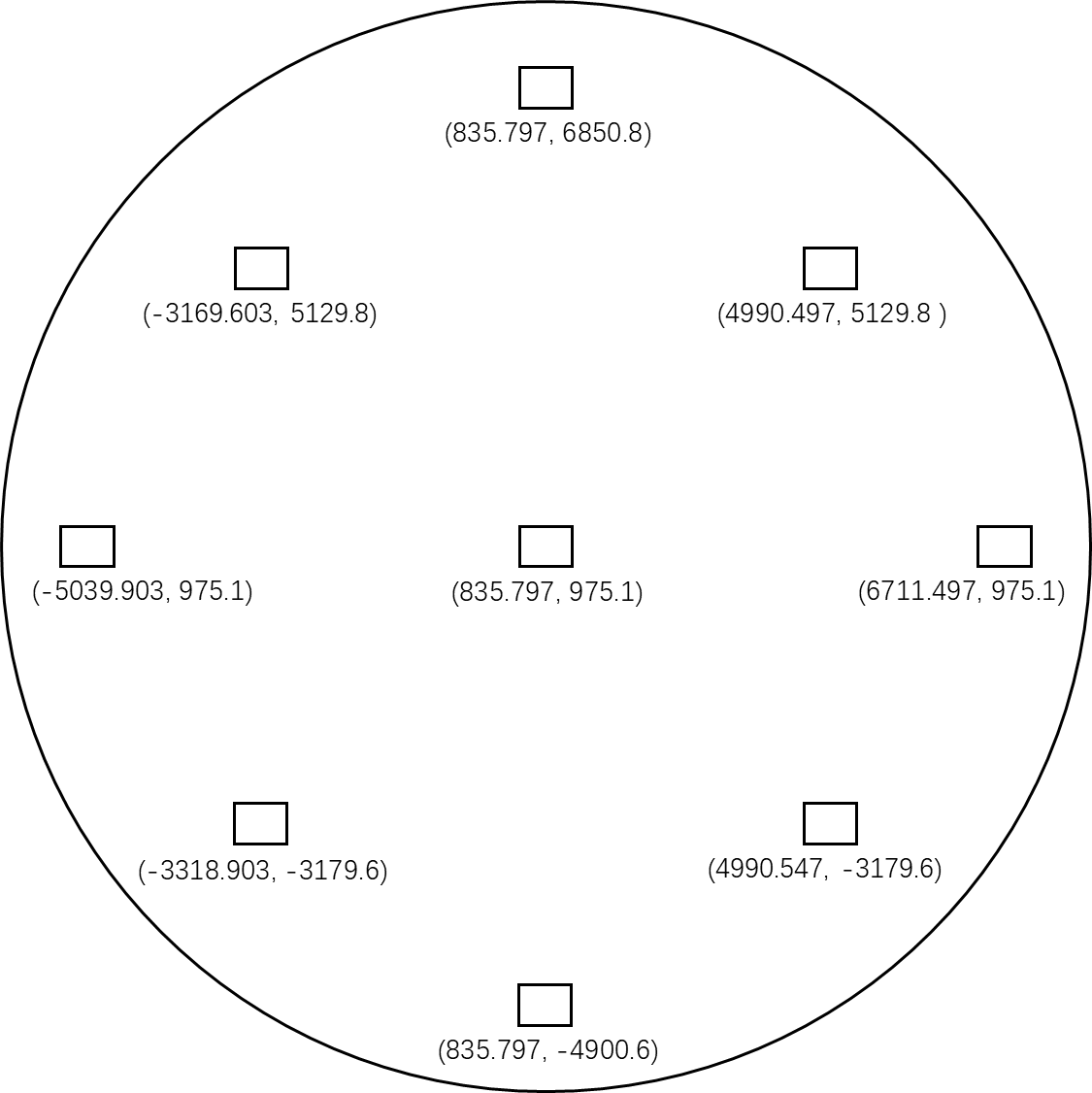


**Fig. S6.** Nine subsampled areas (rectangular area) of the filter, each measuring 480 μm × 640 μm. The total subsampled area accounts for 0.87% of the filter. Please note that this is a distorted illustration. In reality, the squares representing the subsampled areas occupies a much smaller area compared to what is shown in the figure. The coordinates of the center of these subsampled areas are indicated at their respective positions. By utilizing these coordinates, the subsampled areas can always be accurately located.


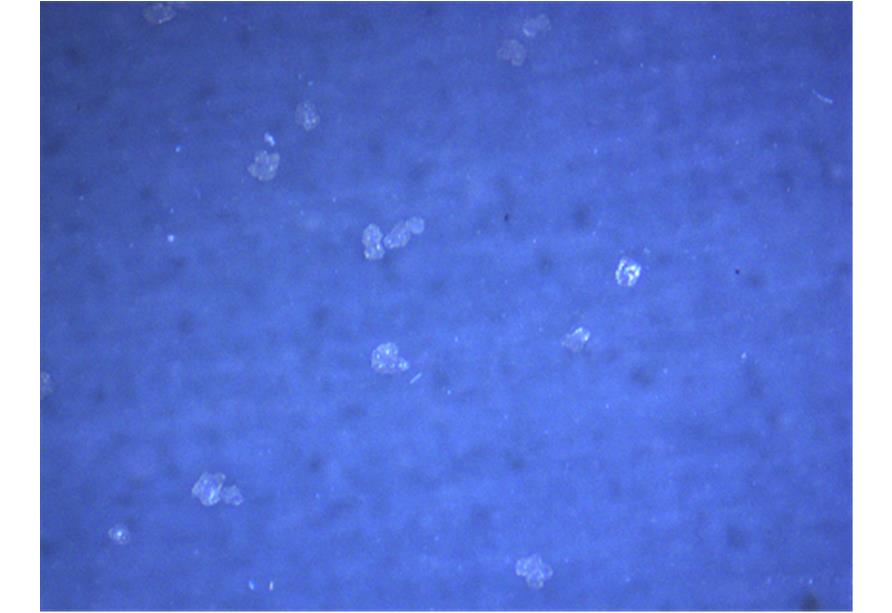


**Fig. S7.** Standard nylon microspheres (average diameter: 15-20 μm) in an optical image of a subsampled area (480 μm × 640 μm) under low magnification (10×). The particle count can be obtained from such an optical image. When instances of particle aggregation were observed, it was possible to determine the number of particles clustered together using high magnification (40×).

**Table S1.** Distribution of Non-MP Particles in 9 subregions for procedural blank samples. The table shows the count of non-MPs particles observed under the same high temperature filtration condition, room temperature filtration condition, and within same steeping commercial nylon teabags experiment design condition across nine different regions. Data are represented as individual replicates.

| Nylon Bulk | Region 1 | 2 | 3 | 4 | 5 | 6 | 7 | 8 | 9 |
| --- | --- | --- | --- | --- | --- | --- | --- | --- | --- |
| Procedural blank Group 1 (Same as High Temp. Experiment condition) | | | | | | | | | |
| Replicate 1 | 0 | 0 | 0 | 0 | 0 | 0 | 0 | 6 | 0 |
| Replicate 2 | 0 | 1 | 0 | 1 | 0 | 2 | 1 | 0 | 0 |
| Replicate 3 | 0 | 0 | 0 | 1 | 0 | 1 | 1 | 0 | 2 |
|  |  |  |  |  |  |  |  |  |  |
| Procedural blank Group 2 (Same as Room Temp. Experiment condition) | | | | | | | | | |
| Replicate 1 | 0 | 1 | 2 | 1 | 1 | 0 | 0 | 0 | 0 |
| Replicate 2 | 0 | 1 | 3 | 0 | 3 | 1 | 1 | 1 | 1 |
| Replicate 3 | 1 | 0 | 1 | 2 | 0 | 1 | 0 | 0 | 1 |
|  |  |  |  |  |  |  |  |  |  |
| Procedural blank Group 3 (Same as Teabag Experiment condition) | | | | | | | | | |
| Replicate 1 | 0 | 0 | 1 | 0 | 1 | 1 | 0 | 0 | 1 |
| Replicate 2 | 1 | 1 | 0 | 0 | 2 | 1 | 0 | 0 | 1 |
| Replicate 3 | 1 | 1 | 0 | 1 | 1 | 1 | 2 | 0 | 1 |
